# The Hyatts’ Anatomical, Pathological, Surgical, and Microscopical Collection

**Published:** 1854-11

**Authors:** Frank H. Hamilton


ART. IV.—*The Hyatts' Anatomical, Pathological, Surgical, and Microscopical Collection.* By FRANK H. HAMILTON, M. D.

I believe that, in a quiet way, the I. C. & D. Hyatts, of New York, are doing more for the improvement of medical science, than any other two men in the United States. I am speaking without any interest in the gentlemen or in their business, except what grows out of an old and very pleasant acquaintance with them, and a hearty good-will toward my fellow men. I think, too, their services have been appreciated and rewarded in some measure. Until these gentlemen began their manufacture and importation of anatomical and pathological specimens, we were obliged to order them from Europe at no inconsiderable expense and delay and no opportunity for selection. It happened, therefore, very naturally, that unless we visited Europe ourselves, we seldom enjoyed the privilege of possessing them. These beautiful models were then, to us, rare and expensive luxuries, but the Hyatts have made them almost common necessities. They are no longer confined to well endowed medical colleges and to men of wealth, but no college to-day is so poor as not to possess some of them; and few physicians are so little informed as not to have seen them, or so dull as not to covet them.

A few years ago these excellent young men were brickmasons, and subsequently they became workers in ornamental stucco. They excelled in every department to which their attention was directed, and having perfected themselves in one branch they always arose easily to the comprehension and mastery of another. Their first efforts in the way of anatomical modeling, consisted in constructing a complete manakin from dissections made by myself, and for which they received a model and a commendatory notice at one of the State Fairs. Subsequently they discovered the process of making models in *papier mache* and plaster—a process unlike Hibert, or Auzoux, and peculiar to themselves, and which I thought possessed some advantages over either of those named. They were more flexible and less liable to fracture. But I am not certain whether they continue to be regarded by themselves as superior.

As their means increased—for these boys had very little money when they began—they gradually enlarged their collection, and a few years since hearing that Thibert was dead, and that his widow wished to sell his collection, the elder brother hurried over to Paris and bought everything at a very large cost. I had visited Thibert's rooms a year or two before, and remember what a divine treat was afforded me. The process by which he made his models was then, and still remains, a secret. The number, and variety, and perfection of the representations, exceeded any thing I had seen either in Florence or in Paris, and I regretted, first, that I was too poor to enjoy them, and next, that my beloved country was much too young to claim or even to hope for their possession. I came home without any thing but a few pieces chosen from the collection, but these young bricklayers have brought us the whole of it.

They have at various times, also, purchased largely of Auzoux, of Guy Ané, and of others, until they have a museum of over 3000 pieces, which they sell at prices varying from one to one thousand dollars each. But their crowning act, and that which has immediately prompted me to speak to you of them, is a recent importation. I have not seen the specimens alluded to, but my confidence in the judgment and honesty of these gentlemen is such that I implicitly receive their own statements for whatever they say to me, and I consider them so nearly my own words that I shall, without consulting them, publish a portion of the letter which I have just received from the senior brother:

"*Dear Doctor:* It is a long time since I have had the pleasure of seeing you, but I wish you could just drop into our rooms and see our present splendid collection of models and preparations, and we think you would say that your old friends have something worth looking at. Among other things which we have just received, we have a *series of natural dissections* by one of the best anatomists in Paris, whose name for reasons which we are not at liberty to give, cannot be announced. These preparations are made by a newly discovered process, and there is nothing in Europe or in this country which can compare with them. The color is preserved; they are quite flexible; there is not the slightest odor, and the various parts are only very little shrunken below their natural dimensions. The best fresh dissections upon the recent subject which I have ever seen, are insignificant when compared with the minuteness and accuracy of these. The injections of the smallest vessels, even of the vessels of the skin, are perfect. It is impossible to give you an adequate idea of their beauty. They must be seen to be appreciated."

I believe that we owe a considerable debt of gratitude to these men, no less, perhaps, than to the enlightened legislators who secured to us our present humane anatomical laws, and I trust, my dear sir, you will think so also.

